# The Impact of Tumor Laterality (Unilateral vs. Bilateral) on Presentation and Management Outcome in Patients with Retinoblastoma

**DOI:** 10.3390/jcm13072146

**Published:** 2024-04-08

**Authors:** Mona Mohammad, Mustafa Mehyar, Hadeel Halalsheh, Reham Shehada, Omar Al Adawi, Jakub Khzouz, Imad Jaradat, Maysa Al-Hussaini, Iyad Sultan, Ibrahim Alnawaiseh, Yacoub A. Yousef

**Affiliations:** 1 Departments of Ophthalmology, King Hussein Cancer Centre (KHCC), Amman 11941, Jordan; mustafamehyar@hotmail.com (M.M.); shehadareham@gmail.com (R.S.); i_nawaiseh@hotmail.com (I.A.); 2 Pediatric Oncology, King Hussein Cancer Centre (KHCC), Amman 11941, Jordan; hadeelhalalsheh@khcc.jo (H.H.); isultan@khcc.jo (I.S.); 3 Pediatric Department, University of Jordan, Amman 11941, Jordan; 4 Pathology, King Hussein Cancer Centre (KHCC), Amman 11941, Jordan; jkhzouz@khcc.jo (J.K.); mhussaini@khcc.jo (M.A.-H.); 5 Radiation Oncology, King Hussein Cancer Centre (KHCC), Amman 11941, Jordan; ijaradat@khcc.jo

**Keywords:** retinoblastoma, unilateral, bilateral, enucleation

## Abstract

**Background:** This study compares the outcomes of managing retinoblastoma between patients with unilateral and bilateral presentations. **Methods:** The study, conducted at the King Hussein Cancer Center in Amman, Jordan, retrospectively analyzed cases of retinoblastoma treated between March 2003 and December 2019. Evaluation criteria included clinical features, disease stage, treatment methods, and overall management outcomes. **Results:** The study comprised 697 eyes from 478 patients with retinoblastoma, with 52% being males. Bilateral disease was observed in 70% of patients, and a family history of retinoblastoma was more prevalent in cases with bilateral disease (20%) compared to those with unilateral disease (4%). Unilateral cases had a median age at diagnosis of 28 months, whereas bilateral cases were diagnosed at a median age of 6 months. Extra-ocular retinoblastoma was detected in 1% of eyes. According to the International Intraocular Retinoblastoma Classification (IIRC), 88% of unilateral cases presented with advanced disease (IIRC group D/E), compared to 46% in bilateral cases. Primary enucleation was performed in 29% of unilateral cases and 16% of bilateral cases (*p*-value 0.0007). Eye salvage rates were 31% in unilateral cases and 68% in bilateral cases (*p*-value < 0.0001). At 120 months of follow-up, 5% of patients died from secondary neoplasms or metastases, 81% were alive, and 14% were lost to follow-up. There was no significant difference in metastasis, secondary neoplasms, or mortality between patients with unilateral and bilateral retinoblastoma. **Conclusions:** This study highlights the nuanced differences in clinical characteristics and outcomes between unilateral and bilateral retinoblastoma, emphasizing the necessity of customized management and early detection strategies. It demonstrates that while bilateral retinoblastoma benefits from earlier detection and has a higher rate of eye salvage, there is no significant difference in metastasis or mortality rates when compared to unilateral cases. The critical roles of primary enucleation in advanced cases, along with effective communication and patient education, are also underscored to improve treatment adherence. Overall, these findings point to the importance of tailored approaches in optimizing outcomes for the diverse patient population affected by retinoblastoma.

## 1. Introduction 

Retinoblastoma (Rb) is recognized as the most frequent malignant intraocular tumor in childhood. The incidence of this malignancy is approximately one in 17,000 live births, translating to about 8000 new cases diagnosed globally each year [1]. The tumor is primarily caused by mutations in the retinoblastoma gene (RB1) located on chromosome 13q14 [2]. Unilateral Rb, which affects only one eye, accounts for approximately 60% to 75% of all Rb cases. In contrast, bilateral Rb makes up the remaining 25% to 40% of cases [3]. The distinction between unilateral and bilateral cases is not merely anatomical but also bears crucial prognostic and therapeutic considerations. 

Retinoblastoma is typically curable through early diagnosis and appropriate management. Treatment strategies depend on factors such as the child’s age, disease stage, and whether the disease is unilateral or bilateral [4]. There are different treatment options which include enucleation, chemotherapy, and various forms of radiation therapy along with focal ophthalmic therapies. Indications for a specific modality or a combination of modalities vary with each patient [5]. For instance, unilateral cases usually present with advanced intraocular disease necessitating enucleation. This approach, while radical, is favored in advanced unilateral cases to avoid the side effects associated with systemic chemotherapy, considering that the other eye is unaffected and retains normal vision. Children with a bilateral disease at diagnosis usually require multimodality treatment (chemotherapy, local therapies). External beam radiotherapy (EBRT) is an option when there is a failure to control the disease in children with bilateral Rb. Enucleation is usually reserved for eyes with recurrent disease and no useful vision, and is rarely performed for both eyes [6].

Therefore, treating bilateral retinoblastoma means a well-planned combination of treatments such as chemotherapy, focal therapies, and radiation. The main aim is to preserve both eyes and maximize vision preservation due to the involvement of both eyes.

The objective of this study is to conduct a comparative evaluation of treatment outcomes in Rb, with a specific focus on comparing the outcome of management between unilateral versus bilateral cases. The aim is to clarify the differences in treatment approaches for these two distinct forms of retinoblastoma, and to offer insights that could improve the treatment decision-making process, ultimately improving patient care and outcomes in this crucial field of pediatric cancer care.

## 2. Materials and Methods

### 2.1. Patients and Study Design

This retrospective study was approved by the institutional review board of King Hussein Cancer Center (KHCC) (ID 02-60-56). The study focused on individuals diagnosed with retinoblastoma at the King Hussein Cancer Center (KHCC) in Amman, Jordan, from March 2003 through December 2019. To be included in the study, patients had to have comprehensive records regarding their initial presentation, the classification stage of their tumor when diagnosed, the treatment approach undertaken, and consistent follow-up information. Patients were excluded from the research if their medical records were incomplete in any of these areas or if they were lost to follow-up, ensuring that the study’s findings were based on thorough and reliable data. Demographic data including sex, nationality, age of onset, laterality, family history of retinoblastoma, stage of disease, follow-up time, and details of primary and adjunctive treatments, eye salvage, mortality, any evidence of metastases and secondary malignancies were obtained from the patients’ medical records and analyzed statistically.

### 2.2. Disease Staging

Each child had a complete ophthalmic examination under general anesthesia, which included binocular indirect ophthalmoscopy with 360-degree indentation, and B-scan ultrasound if there was no fundus view. Fundus imaging was performed using a wide-angle contact fundus camera Ret Cam II camera (Clarity Medical System, Inc., Pleasanton, CA, USA). Magnetic resonance imaging (MRI) scans of the brain and orbits were performed for all patients to look for the presence of extra-ocular extension and/or intracranial tumors (trilateral retinoblastoma). Disease staging was classified according to International Intraocular Retinoblastoma Classification (IIRC) [7] in which each eye was assigned to group A, B, C, D, or E according to the severity of the disease.

### 2.3. Treatment Modalities

Treatment was chosen according to factors that included disease stage, laterality, visual prognosis, child age, and parents’ choice [8]. Eyes in IIRC group A, and some IIRC group B eyes (tumors less than 3 mm thick) were only treated with focal therapy: cryotherapy (MIRA CR 4000) or trans-pupillary thermotherapy (TTT) using 810 nanometers (nm) diode laser, depending on tumor location. For some IIRC group B eyes (tumors 3 mm or more in thickness), eyes in IIRC group C, and some IIRC group D eyes, treatment included either chemo-reduction (CRD) followed by focal therapies, or by intra-arterial chemotherapy (IAC). The selection of treatment was based on the age of patients at the time of treatment, and the feasibility of catheterization during IAC. 

The CRD regimen consisted of combined intravenous chemotherapy (carboplatin-etoposide-vincristine (CVE)), followed by local treatments such as cryotherapy, TTT, or I^125^ radioactive plaque brachytherapy. Focal therapy was applied when needed after the second cycle of systemic chemotherapy. Intravenous chemotherapy was given every 3 to 4 weeks for a total of six to eight cycles [9]. Intra-arterial chemotherapy (IAC) was used in cases of extensive sub-retinal seeding, or for massive tumor recurrence [10].

Primary enucleation was recommended in some unilateral IIRC group D eyes, and for all IIRC group E eyes [11]. In cases of parents’ refusal, systemic chemotherapy followed by focal treatment was proposed as an alternative treatment. Each enucleated eye was examined by an ocular pathologist trained in the evaluation of retinoblastoma to identify high-risk pathological features [12].

The choice of treatment for IIRC group D eyes depended mainly on the status of the other eye: if the other eye did not require treatment with systemic chemotherapy (normal, or IIRC group A or some B eyes) then families were offered either primary enucleation or trial of globe salvage with CRD. If the other eye required systemic chemotherapy (some IIRC B, IIRC C, or D), then patients were treated with CRD followed by focal consolidation therapy as described above.

Intravitreal chemotherapy with melphalan was reserved for eyes with persistent vitreous seeds, which responded poorly to systemic chemotherapy and/or IAC [13].

Iodine (I^125^) plaque brachytherapy was used in cases of localized recurrence of tumor activity if parents refused enucleation [14].

Eyes that failed the above-mentioned treatment options were secondarily enucleated [15].

Globe salvage was defined as the absence of tumor activity or recurrence, and the absence of active vitreous or subretinal seeds after a minimum of one year follow-up visit with no evidence of metastasis without the need for enucleation or external beam radiation therapy (EBRT).

### 2.4. Statistical Analysis

In this study, descriptive statistics were described with parametric and non-parametric statistics as needed. The statistical significance was set at *p*-value *p* < 0.05. This approach allowed for the identification of meaningful differences and correlations within the data, ensuring that the conclusions drawn from the study were based on statistically significant findings. Multivariate analysis was done using a logistic regression model.

A significance criterion of *p* ≤ 0.05 was used in the analysis. All analyses were performed using SAS version 9.4 (SAS Institute Inc, Cary, NC, USA).

## 3. Results

### 3.1. Patient Demographics (Table 1)

Between March 2003 and December 2019, the KHCC Rb service treated 478 children with Rb; 249 (52%) patients were males, and 335 (70%) patients had bilateral disease. Non-Jordanian patients were more frequently diagnosed with bilateral retinoblastoma than Jordanians, with occurrences of 79% and 55%, respectively. A family history of Rb was present in six (4%) of 249 unilateral cases and in 66 (20%) of bilateral cases (*p*-value ˂ 0.0001). The median age at diagnosis was 6 and 28 months for patients with bilateral and unilateral Rb, respectively. Of 813 eyes with Rb, 116 were enucleated before referral to our service; therefore, 697 affected eyes were treated during the study period.

**Table 1:** Demographics, Presentation, Primary Management, and Management Outcome.

		Patients with Unilateral RB	Patients with Bilateral RB	All Study Population	*p* Value
		*n* (%)	*n* (%)	*n* (%)	
Number of patients		143 (30%)	335 (70%)	478	
Number of affected eyes ^a^		143 (21%)	554 (79%)	697	
Age at diagnosis (Months) Mean, Median (Range)		31, 28, (0.25–252)	4, 6, (0.25–40)	16, 12, (0.25–252)	
Male	Sex	76 (31%)	173 (69%)	249 (52%)	0.761
Female		67 (29%)	162 (71%)	229 (48%)	
Negative	Family history	137 (34%)	269 (80%)	406 (66%)	<0.0001
Positive		6 (8%)	66 (20%)	72 (15%)	
Jordanian	Nationality	82 (45%)	101(55%)	183 (38%)	<0.0001
Non-Jordanian		61 (21%)	234 (79%)	295 (62%)	
A, B, C	Tumor stage at diagnosis	15 (10%)	294 (53%)	309 (44%)	<0.0001
D, E		126 (88%)	256 (46%)	382 (55%)	
Extraocular		2 (2%)	4 (1%)	6 (1%)	
Enucleation	Primary treatment	42 (29%)	90 (16%)	132 (19%)	0.0007
Conservative therapy		101 (71%)	464 (84%)	565 (81%)	
Yes	Eye Globe Salvage (for attempted salvage)	45 (31%)	376 (81%)	421(75%)	<0.0001
No		56 (55%)	88 (19%)	144 (25%)	
Secondary enucleation	Treatment failure	52 (51%)	62 (13%)	114 (20%)	0.0013
EBRT		3 (2%)	13 (4%)	16 (3%)	
EBRT + Enucleation		1 (1%)	13 (4%)	14 (2%)	
Metastasis		8 (6%)	14 (4%)	22 (4%)	0.4652
Secondary neoplasms		0 (0%)	4 (1.5%)	4 (1%)	0.167
Mortality		8 (6%)	16 (5%)	24 (5%)	0.8193

^a^ The number of patients was 478, with 813 total affected eyes. Of these, 116 were enucleated before referral to our center, and six (two with unilateral and four with bilateral disease) eyes presented with extraocular disease.

### 3.2. Tumor Characteristics, Management, and Outcomes (Table 1 and Table 2)

Compared to patients with bilateral Rb, those with unilateral Rb presented with more advanced disease: for the unilateral group, 126/143 of eyes (88%) were in IIRC group D/E compared to 256/554 (46%) in bilateral patients. Primary enucleation was performed for 29% (*n* = 42/143) of eyes in patients with unilateral disease and for 16% (*n* = 90/554) of eyes in patients with bilateral disease (*p*-value 0.0007). The overall eye salvage rates were 31% for patients with unilateral disease and 68% for patients with bilateral disease (*p*-value < 0.0001). Of the 478 patients with Rb treated at KHCC, six (1%) had bilateral enucleation: four patients had one eye previously enucleated before coming to KHCC, the other two had bilateral IIRC group E Rb with extraocular extension necessitating bilateral enucleation, as shown in Table 1.

**Table 2:** The Impact of Laterality (Unilateral versus Bilateral) in Patients with Retinoblastoma on Management and Eye Salvage Rates.

	Unilateral (143 Eyes for 143 Patients) *n* (%)				Bilateral (554 Eyes for 335 Patients) *n* (%)				*p* Value
	Number	Primary Enucleation	Conservative Therapy	Eye Salvage	Number	Primary Enucleation	Conservative Therapy	Eye Salvage	
Total	143	42 (29%)	101	45 (31%)	554	90 (16%)	464	376 (68%)	<0.0001
IIRC group A	1	0 (0%)	1	1(100%)	40	0 (0%)	40	39 (98%)	0.872
IIRC group B	3	0 (0%)	3	3 (100%)	103	0 (0%)	103	96 (93%)	0.640
IIRC group C	11	4 (36%)	7	6 (55%)	151	4 (3%)	147	138 (91%)	0.002
IIRC group D	111	21 (19%)	90	35 (32%)	198	32 (16%)	166	102 (52%)	0.0008
IIRC group E	15	15 (100%)	0	0 (0%)	58	50 (86%)	8	1 (2%)	1.00
Extra ocular	2	2 (100%)	0	0 (0%)	4	4 (100%)	0	0 (0%)	1.00
	**Unilateral (143 eyes for 143 patients)**				**Bilateral (554 eyes for 335 patients)**				
Neoadjuvent IVC	101 (71%)				234 (70%)				0.478
Focal therapy	101 (71%)				464 (84%)				0.0004
Intra Arterial Chemotherapy	2 (1%)				13 (2%)				0.376
Intra Vitreal Chemotherapy	4 (3%)				17 (3%)				0.563
EBRT	4 (3%)				36 (7%)				0.042
Radioactive plaque	1(1%)				12 (2%)				0.418
Primary enucleation	42 (29%)				90 (16%)				0.0006
Secondary enucleation	52 (36%)				62 (11%)				<0.0001
Adjuvant IVC	16 (11%)				33 (10%)				0.781

Between 2003 and 2009, 142 patients underwent treatment, comprising 55 with unilateral retinoblastoma (Rb) and 87 with bilateral Rb (174 eyes). The overall eye salvage rate was 47%, with 31% for unilateral Rb (*n* = 17/55) and 53% for bilateral Rb (*n* = 92/174). From 2010 to 2019, 336 patients were treated, including 88 with unilateral Rb and 248 with bilateral Rb (496 eyes). The overall eye salvage rate was 53%, with 32% for unilateral Rb (*n* = 28/88) and 57% for bilateral Rb (*n* = 284/248). There was no statistically significant difference in eye salvage rates between the early (2003–2009) and late (2010–2019) periods for either unilateral or bilateral Rb (all *p* > 0.05). The eye salvage rate between 2010–2019 was slightly higher but not statistically significant) than between 2003–2009 because of implementing new treatments like intra-arterial chemotherapy, intra-vitreal chemotherapy, and radioactive plaque therapy. These three modalities (Table 2) were given for patients after 2010.

In a logistic regression analysis examining factors affecting the ‘salvage’ outcome in patients with retinoblastoma, significant findings were observed for both tumor laterality and the IIRC stage. Patients with bilateral tumors were found to have significantly higher odds (Odds Ratio: 0.449, 95% CI: 0.272–0.740, *p* = 0.0017) of achieving a ‘salvage’ outcome compared to those with unilateral tumors. Additionally, patients classified in the lower IIRC stages (A, B, C) showed significantly higher odds (Odds Ratio: 11.379, 95% CI: 6.543–19.787, *p* < 0.0001) of a ‘salvage’ outcome compared to those in the advanced stages (D, E), highlighting the importance of early disease stage in favorable outcomes. These results underscore the significant impact of tumor laterality and early-stage disease on the management and prognosis of retinoblastoma (Table 3).

Out of thirty-six patients (40 eyes) who received EBRT, four (all with bilateral Rb) developed second neoplasms (three had osteosarcoma and one had liposarcoma). Of these four patients, three died of their disease, and one is currently alive and free of metastases. In a 120-month median follow-up period, 24 (5%) patients died of second neoplasms (*n* = 3) or metastases (*n* = 21); 81% were alive; and 14% were lost to follow-up.

Of interest, the issue of treatment abandonment was encountered with five patients (1%). Among these cases, two were Jordanian children with unilateral group E eyes; parents refused enucleation and sought treatment outside the country. Abroad, enucleation was offered and rejected by parents, and both presented later with extraocular disease, central nervous system (CNS) involvement, and bone marrow metastasis. One Jordanian patient with unilateral group D eyes, and one non-Jordanian patient with bilateral Rb (one eye in group E and the other in group C) refused all types of treatment (enucleation or chemotherapy), and both presented one year later with extraocular disease and bone marrow metastasis. The fifth patient was a non-Jordanian child with bilateral Rb (one eye had extensive optic nerve invasion in MRI) who refused treatment and went back to his country, and was completely lost to follow-up. The four patients who returned after treatment abandonment died of distant metastasis. 

## 4. Discussion

The primary objectives of treating retinoblastoma, prioritized as preserving life, the eye itself, and vision, guided our treatment approach. This approach was tailored to each patient, considering factors such as whether the tumor affected one or both eyes, the stage and location of the tumor, the potential for vision, and family preferences [16]. Our study focused on examining how these treatment outcomes were influenced by the condition of the patient’s other eye.

Similar to other studies, the median age at diagnosis in our patients with bilateral disease was significantly lower than that of patients with unilateral disease (6 months vs. 28 months) [17]. However, for unilateral cases our results showed a more delayed median age of diagnosis as compared to other studies [18]. This indicates that our patients presented later with more advanced disease.

Our patient population did not exhibit a sex predilection, with a nearly equal distribution of males (52%) and females (48%), aligning with findings from several previous studies [19]. However, contrary to this trend, research from India has indicated a male predominance in retinoblastoma cases, which has been attributed to the lesser attention given to female children in that region [20].

While our research indicated a higher incidence of bilateral retinoblastoma (70% bilateral vs. 30% unilateral), studies from India [21] and the United States [22] have shown a greater prevalence of unilateral cases. The variance in our results might stem from a referral bias, especially among non-Jordanian patients, where 62% of our study’s demographic were non-Jordanians and 79% of these had bilateral Rb. Referral bias elucidates the notable disparity in the laterality of retinoblastoma between Jordanian and non-Jordanian patients, with a particular impact on treatment and referral patterns. Non-Jordanian children with unilateral retinoblastoma often receive treatment, typically enucleation within their local healthcare settings. On the other hand, bilateral retinoblastoma cases, which necessitate a more comprehensive and specialized treatment regimen, including the potential for limb-sparing therapies and advanced oncologic care, result in these patients being more frequently referred to higher-tier medical centers in Jordan. This distinction in treatment and referral practices based on the condition’s laterality significantly contributes to the observed difference in the proportion of bilateral versus unilateral cases between Jordanian and non-Jordanian groups, highlighting how local treatment capabilities and the complexity of medical needs influence patient referrals across borders. Moreover, larger family sizes in Jordan, which often include multiple members with the condition, might also explain the higher bilateral cases in our study, as 55% of Jordanian patients had bilateral Rb [23].

Among patients with intraocular retinoblastoma, a higher incidence of advanced-stage presentations (IIRC groups D or E) was observed in unilateral cases (88%) as opposed to bilateral ones (46%), as shown in Table 1. This could be attributed to the fact that once a patient is found to have Rb in one eye, examination for the other eye allows the detection of a tumor at an earlier stage. In addition, patients with bilateral retinoblastoma tend to seek medical attention sooner due to the manifestation of visual symptoms in both eyes, unlike those with unilateral retinoblastoma, who often have one unaffected eye with normal vision. Furthermore, bilateral cases are often hereditary, leading to their earlier identification through proactive screening in children considered at risk [24].

Our results showed that the overall eye salvage rates were 31% for patients with unilateral disease and 68% for patients with bilateral disease. In unilateral retinoblastoma, primary enucleation is more readily accepted due to the advanced stage of the affected eye and the existence of a healthy fellow eye. Similarly, secondary enucleation following unsuccessful treatment is more acceptable when the alternate eye is unaffected. In contrast, for bilateral retinoblastoma, there is considerable resistance to bilateral enucleation amidst disease progression [8]. This variation in parental willingness to accept enucleation aligns with our findings of higher globe salvage rates in bilateral cases compared to unilateral ones. Moreover, patients with unilateral retinoblastoma often presented with a more advanced stage of the disease, necessitating enucleation as the primary treatment approach.

Our findings indicate no significant difference in the incidence of metastasis, secondary neoplasms, or disease-related mortality between unilateral and bilateral retinoblastoma patients. This is a crucial observation, suggesting that the higher globe salvage rates in bilateral cases do not compromise the overall survival and safety of the children.

External beam radiotherapy (EBRT) is more frequently used in cases of bilateral retinoblastoma [25], with 4% of bilateral cases undergoing EBRT, in contrast to just 2% in unilateral cases. In situations where unilateral Rb progresses, enucleation is often deemed a wiser choice to minimize radiation exposure. Conversely, for bilateral RB, the prospect of bilateral enucleation is challenging to accept, leading to a greater inclination towards using radiation therapy despite the associated risks of secondary malignancies [26].

The refusal of retinoblastoma treatment by parents, as highlighted by the five cases in our study, underscores a critical challenge in the management of retinoblastoma. In these instances, treatment refusal led to advanced disease progression and metastasis, emphasizing the need for effective communication between healthcare providers and families. It is important to address cultural, informational, and psychological barriers that might influence such decisions. This highlights the necessity for comprehensive patient education and the development of supportive strategies to encourage adherence to life-saving treatments in pediatric cancer care [27].

A study from Thailand found that families often find primary enucleation unacceptable. Insisting on this treatment can lead to refusal, abandonment, and loss to follow-up. Offering globe salvage treatment in these cases helps keep families engaged and makes secondary enucleation more palatable [17]. A Malaysian study noted widespread refusal of enucleation upon recommendation [28].

A prospective analysis performed in by the Global Retinoblastoma Study Group showed that during the year 2017, enucleation rates for Rb decreased with rising national income levels: 74% in low-income countries, 67% in lower-middle-income countries, 62% in upper-middle-income countries, and 59% in high-income countries [29]. Jordan, classified as a lower-middle-income country, demonstrates a significantly lower overall enucleation rate of 49% (Table 4).

However, more recent studies showed a trend in more globe salvage rates in high-income countries [30,31]. A critical breakthrough in the treatment of retinoblastoma has been the enhancement of localized drug administration methods. Techniques such as intravitreal chemotherapy [13,32] and intra-ophthalmic artery chemosurgery [10,33] have substantially raised the chances of saving the affected eyes. These approaches have achieved remarkable success in the management of the disease’s advanced stages, which traditional systemic chemotherapy and external radiation therapy have not effectively addressed. 

In a meta-analysis conducted by Daniels and colleagues, to evaluate the impact of various intravenous chemotherapy regimens on the success rates of saving the eye in retinoblastoma cases, they found that for eyes with diffuse vitreous seeds, an optimized regimen that combines “standard” intravenous chemotherapy with additional treatments like intravitreal chemotherapy significantly outperformed the standard approach (Odds Ratio = 2.4 (95% Confidence Interval: 1.3–4.7); *p* = 0.004) [34].

Moreover, the advancement in healthcare delivery in regions with high-income countries has led to exceedingly high survival rates, along with the preservation of the eye and vision. However, such positive outcomes are not mirrored in healthcare systems within middle-income and low-income areas. An analysis segmented by national income showed that the failure to save the eye was observed in 25.2% of cases in high-income countries, 29.8% in upper-middle-income countries, and 27.4% in low-middle-income countries [30].

Our results showed that the eye salvage rate between 2010–2019 (53%) was slightly higher, but not statistically significant, than between 2003–2009 (47%), because of implementing new treatments like intra-arterial chemotherapy, intra-vitreal chemotherapy, and radioactive plaque therapy. These three modalities were given for our patients after 2010. While the current rates of eye preservation in Jordan may not match those observed in high-income countries, the ongoing introduction of innovative retinoblastoma treatment methods is expected to significantly enhance these rates over time. Future research emanating from Jordan is anticipated to underscore the positive impact of these advanced treatment modalities on improving the outcomes of eye preservation in Rb.

This study’s limitations include its retrospective design, which may introduce selection and recall biases. The generalizability of the findings may be limited due to the specific demographic and treatment modalities at our center. Additionally, the relatively small sample size, particularly for certain subgroups, may affect the robustness of the statistical analysis. Furthermore, long-term follow-up data was not available for all patients, potentially impacting the assessment of outcomes like late metastasis or long-term survival. These factors should be considered when interpreting the study’s findings.

In conclusion, the management of retinoblastoma is complex, requiring a patient-specific approach. Key findings include earlier detection and higher globe salvage rates in bilateral cases, and no significant difference in metastasis or mortality between unilateral and bilateral cases. The study also emphasizes the importance of considering cultural sensitivities and family preferences, especially in the context of treatment acceptance. Effective communication and patient education are crucial in ensuring adherence to treatment and improving outcomes in pediatric retinoblastoma care.

In cases of advanced unilateral retinoblastoma (IIRC groups D and E), primary enucleation is a critical, life-saving measure. It not only effectively cures the disease but also plays a pivotal role in saving the patient’s life, reducing the metastasis risk, and decreasing the need for frequent follow-ups. Additionally, it helps in avoiding the potential side effects associated with systemic chemotherapy or intra-arterial chemotherapy (IAC).

These findings offer insights into tailored management strategies for Rb, emphasizing the need for early detection, patient education, and considering individual disease characteristics in treatment planning.

## Figures and Tables

**Table 3:** Impact of Tumor Laterality and IIRC Stage on Salvage Outcome in Retinoblastoma: A Logistic Regression Analysis.

Effect	Odds Ratio	95% Wald Confidence Limits		*p*-Value
**Laterality (Bilateral vs. Unilateral)**	0.449	0.272	0.740	0.0017
**IIRC (A,B,C vs D,E)**	11.379	6.543	19.787	<0.0001

**Table 4:** Enucleation rate for Rb in relation to national income level.

National Income Level	Enucleation Rate According to Global Retinoblastoma Study Group [29]	Enucleation Rate According to American Joint Committee on Cancer—Ophthalmic Oncology Task Force [30]
Low income	74%	N/A
Lower-middle income	67 %	27.4%
Upper-middle income	62%	29.8%
High income	59%	25.2%
Our results (Jordan) *	49% (Unilateral 69%, Bilateral 32%)	

* Jordan is classified as a lower-middle-income country.

## Data Availability

Data are available on reasonable request on demand to the corresponding authors.
